# London Prices of Drugs.—June 24

**Published:** 1814-07

**Authors:** 


### LONDON PRICES OF DRUGS.—June 24.

*(To be continued regularly.)*

| | £ | s | d. | £. | s. | d. | per |
|---|---|---|---|---|---|---|---|
| Aloes Barbadoes, from | 20 | 10 | 0 to | 21 | 0 | 0 | C. |
| ——Cape | 10 | 0 | 0.. | 11 | 0 | 0 | — |
| ——Succotrina | 20 | 0 | 0.. | 0 | 0 | 0 | — |
| ——Epatica or E.I. | 14 | 0 | 0.. | 16 | 0 | 0 | — |
| Angelica Root | 9 | 10 | 0.. | 9 | 15 | 0 | — |
| Alkanet Root | 6 | 0 | 0.. | 0 | 0 | 0 | — |
| Antimony Crude | 2 | 15 | 0.. | 3 | 0 | 0 | — |
| Aqua Fortis | S. | 0 | 8.. | D. | 1 | 1 | lb. |
| Arrow Root fine | 0 | 2 | 0.. | 0 | 3 | 6 | — |
| ——ordinary | 0 | 1 | 0.. | 0 | 1 | 6 | — |
| Arsenic Red | 5 | 0 | 0.. | 0 | 0 | 0 | C. |
| ——White | 2 | 17 | 0.. | 3 | 0 | 0 | — |
| Balsam Capivi | 0 | 6 | 0.. | 0 | 6 | 3 | lb. |
| ——Peru | 1 | 0 | 0.. | 1 | 1 | 0 | — |
| ——Tolu | 0 | 14 | 0.. | 0 | 14 | 6 | — |
| Bark Jesuits', Com. | 0 | 3 | 6.. | 0 | 4 | 0 | — |
| ——Second | 0 | 6 | 0.. | 0 | 7 | 0 | — |
| ——Quil or best | 0 | 8 | 0.. | 0 | 10 | 0 | — |
| ——Red | 0 | 7 | 0.. | 0 | 10 | 0 | — |
| ——Yellow | 0 | 4 | 6.. | 0 | 5 | 0 | — |
| Borax refined E.I. | none. | | | | | | |
| ——English | 0 | 3 | 9.. | 0 | 4 | 0 | lb. |
| ——unrefined or Tinc. | 10 | 0 | 0.. | 11 | 0 | 0 | C. |
| Camphire refined | 0 | 8 | 9.. | 0 | 9 | 0 | lb. |
| ——unrefined | 28 | 0 | 0.. | 32 | 0 | 0 | C. |
| Cantharides | 8 | 10 | 0.. | 0 | 9 | 0 | lb. |
| Cardamoms (best) | 0 | 7 | 0.. | 0 | 12 | 0 | — |
| Cassia Buds | 36 | 0 | 0.. | } | 0 | 0 | C. |
| ——Fistula W.I. | 6 | 10 | 0.. | in bond | | | — |
| ——Lignea | 25 | 0 | 0.. | } | 0 | 0 | — |
| Castoreum American | 2 | 10 | 0.. | 2 | 15 | 0 | lb. |
| ——Russia | 11 | 0 | 0.. | 0 | 0 | 0 | — |
| Castor Oil per bottle 1½ lb. | 0 | 3 | 9.. | 0 | 4 | 3 | bo |
| Cocculus Indicus | 6 | 0 | 0.. | 7 | 0 | 0 | C. |
| Colocynth Turkey | 0 | 5 | 0.. | 0 | 5 | 6 | lb. |
| Columbo Root | 4 | 15 | 0.. | 6 | 0 | 0 | C. |
| Cream of Tartar | 6 | 10 | 0.. | 7 | 10 | 0 | — |
| Gallangal East-India | 5 | 0 | 0.. | 0 | 0 | 0 | C. |
| Gentian Root | 3 | 5 | 0.. | 3 | 6 | 0 | — |
| Ginseng | 0 | 1 | 6.. | 0 | 0 | 0 | lb. |
| Grains of Guinea | 6 | 6 | 0.. | 6 | 10 | 0 | C. |
| Gum Ammo. Drop | 30 | 0 | 0.. | 0 | 0 | 0 | — |
| ——Lump | 8 | 0 | 0.. | 9 | 0 | 0 | — |
| ——Animi | 5 | 10 | 0.. | 7 | 10 | 0 | — |
| ——Arabic E.I. | 2 | 0 | 0.. | 4 | 0 | 0 | — |
| ——Turkey Fine | 10 | 10 | 0.. | 14 | 14 | 0 | — |
| ——Barbary | 7 | 7 | 0.. | 8 | 0 | 0 | — |
| ——Assafœtida | 10 | 0 | 0.. | 15 | 0 | 0 | — |
| ——Benjamin | 8 | 10 | 0.. | 35 | 0 | 0 | — |
| ——Cambogium | 20 | 0 | 0.. | 26 | 0 | 0 | — |
| ——Copal scraped | 0 | 3 | 0.. | 0 | 4 | 6 | lb. |
| ——Galbanum | 16 | 0 | 0.. | 20 | 0 | 0 | C. |

| | £. | s. | d. | £. | s. | d. | per |
|---|---|---|---|---|---|---|---|
| Gum Guaiacum, from | 0 | 2 | 6 to | 0 | 3 | 0 | lb. |
| ——Mastic | 0 | 4 | 6.. | 0 | 4 | 9 | — |
| ——Myrrh | 15 | 0 | 0.. | 18 | 0 | 0 | C. |
| ——Olibanum | 6 | 0 | 0.. | 7 | 0 | 0 | — |
| ——Oppoponax | 80 | 0 | 0.. | 0 | 0 | 0 | — |
| ——Sandrac | 9 | 0 | 0.. | 10 | 0 | 0 | — |
| ——Seneca Garbled | 5 | 2 | 0.. | 5 | 5 | 0 | — |
| ——Tragacanth | 36 | 0 | 0.. | 38 | 0 | 0 | — |
| Jalap | 0 | 5 | 0.. | 0 | 5 | 3 | lb. |
| Ipecacuanha | 1 | 0 | 0.. | 1 | 1 | 0 | — |
| Isinglass Book | 0 | 10 | 6.. | 0 | 11 | 0 | — |
| ——Leaf | 0 | 10 | 6.. | 0 | 11 | 0 | — |
| ——Long Staple | 0 | 10 | 6.. | 0 | 11 | 0 | — |
| ——Short Staple | 0 | 10 | 6.. | 0 | 11 | 0 | — |
| Manna Flakey | 0 | 5 | 3.. | 0 | 5 | 6 | — |
| ——Sicily in Sorts | 0 | 4 | 0.. | 0 | 4 | 3 | — |
| Musk China | 0 | 18 | 0.. | 1 | 0 | 0 | oz. |
| Nux Vomica | 3 | 0 | 0.. | 3 | 10 | 0 | C. |
| Oil of Vitriol | 0 | 0 | 3½ | 0 | 0 | 0 | lb. |
| Opium East-India | 1 | 2 | 0.. | 1 | 3 | 0 | — |
| ——Turkey | 1 | 9 | 0.. | 1 | 10 | 0 | — |
| Pink Root | 0 | 4 | 0.. | 0 | 4 | 6 | — |
| Quicksilver | 0 | 4 | 10.. | 0 | 0 | 0 | — |
| Rhubarb East-India | 0 | 6 | 0.. | 0 | 10 | 0 | — |
| ——Russia | 0 | 18 | 0.. | 1 | 0 | 0 | — |
| Saffron Spanish | 2 | 5 | 0.. | 2 | 10 | 0 | — |
| ——French | 1 | 15 | 0.. | 2 | 0 | 0 | — |
| Sago | 7 | 0 | 0.. | 8 | 0 | 0 | C. |
| Sal. Ammoniac | 10 | 10 | 0.. | 11 | 0 | 0 | — |
| Sarsaparilla | 0 | 3 | 0.. | 0 | 3 | 6 | lb. |
| Sassafras | 7 | 0 | 0.. | 8 | 0 | 0 | T. |
| Scammony Aleppo | 1 | 6 | 0.. | 1 | 7 | 0 | lb. |
| ——Smyrna | 0 | 10 | 0.. | 0 | 12 | 0 | — |
| Senna | 0 | 3 | 0.. | 0 | 3 | 6 | — |
| Seeds Anni Alicant | 6 | 0 | 0.. | 6 | 10 | 0 | C. |
| ——Coriander English | 1 | 10 | 0.. | 1 | 16 | 0 | — |
| ——Cummin | 7 | 0 | 0.. | 7 | 10 | 0 | — |
| ——Fenugreek | 1 | 10 | 0.. | 1 | 13 | 0 | — |
| Shellack | 7 | 10 | 0.. | 10 | 0 | 0 | — |
| Sticklack | 6 | 0 | 0.. | 8 | 0 | 0 | — |
| Snake Root | 0 | 0 | 0.. | 0 | 0 | 0 | lb. |
| Soap Castile or Spanish | 12 | 0 | 0.. | 14 | 0 | 0 | C. |
| Spermaceti refined | 0 | 2 | 0.. | 0 | 0 | 0 | lb. |
| Tamarinds West-India | 8 | 10 | 0.. | 9 | 0 | 0 | C. |
| Tapioca Lisbon | 0 | 0 | 8.. | 0 | 1 | 0 | lb. |
| Turmeric Bengal | 5 | 10 | 0.. | 0 | 0 | 0 | C. |
| ——China | 6 | 10 | 0.. | 7 | 0 | 0 | — |
| ——West-India | 8 | 0 | 0.. | 9 | 0 | 0 | — |
| Verdigris Wet | 0 | 3 | 6.. | 0 | 0 | 0 | lb. |
| ——Dry | 0 | 4 | 6.. | 0 | 0 | 0 | — |
| ——Crystallized | 0 | 8 | 0.. | 0 | 0 | 0 | — |
| Vitriol Roman | 0 | 0 | 7.. | 0 | 0 | 0 | — |
| ——Foreign white | 2 | 18 | 0.. | 3 | 0 | 0 | C. |

*Price of Vials per Gross.*—8 oz. 70s.—6 oz. 58s.—4 oz. 47s.—3 oz. 43s.—2 oz. 36s.—1 oz. 30s. —½ oz. 24s.

### OBSERVATIONS.

At recent Sales of Merchandize by Auction, Mr. J. Twemlow sold, on the 22d of June, 12 chests Tolpha Manna, 2s. 6d. to 2s. 7d. per lb.—19 bags Sago, 105s. per cwt.—6 casks Cream of Tartar, 6l. 15s. to 7l. 12s. 6d. per cwt.—42 chests Bologna Argol, 5l. 5s. to 5l. 17s. 6d. per cwt.

